# Supplementary material for: Predicting visual function by interpreting a neuronal wiring diagram
Source: Nature. 2024 Oct 2;634(8032):113–23. doi: 10.1038/s41586-024-07953-5 (PMC11446822; doi:10.1038/s41586-024-07953-5)
Supplement: Supplementary file 6 — CRF and ERF predictions for individual TmY4 and TmY9 cells. Analogous to Supplementary Data 3, but for TmY target types. Shown are the top four monosynaptic pathways, the strongest pathway passing through each of the top ten intermediary types (ranking from Extended Data Fig. 7), and the trisynaptic pathway Tm1–TmY–Dm3–TmY (see the section entitled Prediction of spatial normalization). [file 41586_2024_7953_MOESM6_ESM.zip › DataS4/TmY9qΓèÑ/720575940626564997.pdf]

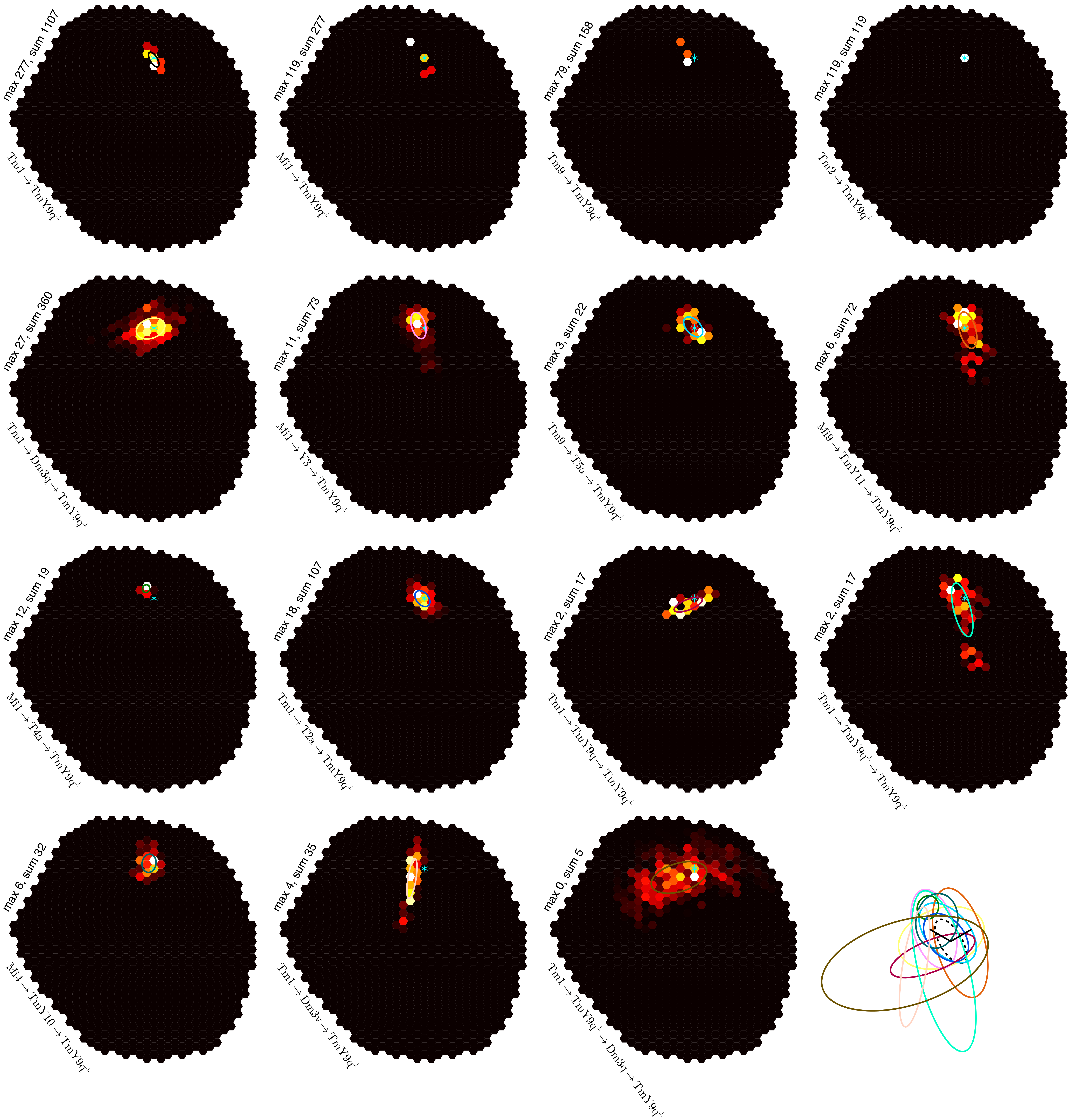
max 277, sum 1107
Tm1 → TmY9q⊥
max 119, sum 277
Mi1 → TmY9q⊥
max 79, sum 158
Tm9 → TmY9q⊥
max 119, sum 119
Tm2 → TmY9q⊥
max 27, sum 360
Tm1 → Dm3q → TmY9q⊥
max 11, sum 73
Mi1 → Y3 → TmY9q⊥
max 3, sum 22
Tm9 → T5a → TmY9q⊥
max 6, sum 72
Mi9 → TmY11 → TmY9q⊥
max 12, sum 19
Mi1 → T4a → TmY9q⊥
max 18, sum 107
Tm1 → T2a → TmY9q⊥
max 2, sum 17
Tm1 → TmY9q → TmY9q⊥
max 2, sum 17
Tm1 → TmY9q⊥ → TmY9q⊥
max 6, sum 32
Mi4 → TmY10 → TmY9q⊥
max 4, sum 35
Tm1 → Dm3v → TmY9q⊥
max 0, sum 5
Tm1 → TmY9q⊥ → Dm3q → TmY9q⊥
